# Beyond the formal curriculum: unveiling the pathway from hidden curriculum to professional identity through learning engagement in nursing education

**DOI:** 10.1186/s12909-026-09332-2

**Published:** 2026-05-02

**Authors:** Chenglei Wu, Xinyue Niu, Huiqi Chen, Yi Qiu, Yanan Shi, Qin Shen

**Affiliations:** The College of Nursing, Zhejiang Chinese Medical University, Hangzhou, 310053 China

**Keywords:** Nursing education, Hidden curriculum, Learning engagement, Professional identity, Undergraduate nursing students

## Abstract

**Background:**

Strengthening professional identity is crucial to mitigate nursing students’ attrition and ensure a sustainable workforce. While the hidden curriculum and learning engagement are believed to influence this identity, their interrelationships remain unclear.

**Aims:**

This study aims to explore the relationship between hidden curriculum and professional identity and examine whether learning engagement mediates the relationship between hidden curriculum and professional identity.

**Methods:**

A cross-sectional study was conducted among 623 undergraduate nursing students from two medical colleges in China. The general information questionnaire, Hidden Curriculum Evaluation Scale in Nursing Education, Utrecht Work Engagement Scale-Student, and Professional Identity Scale for Nursing Students were used for data collection, and IBM SPSS 26.0 and PROCESS macro (Model 4) were used for statistical analysis.

**Results:**

Hidden curriculum, learning engagement, and professional identity were significantly positively correlated. Learning engagement partially mediated the relationship between hidden curriculum and professional identity, accounting for 44.13% of the total effect.

**Conclusions:**

Nursing educators should proactively develop the hidden curriculum and implement strategies to boost learning engagement, thereby fostering students’ professional identity and reducing future workforce attrition.

**Supplementary Information:**

The online version contains supplementary material available at 10.1186/s12909-026-09332-2.

## Introduction

The shortage of nurses and high turnover rates remain persistent challenges for health systems worldwide [1]. According to The State of the World’s Nursing 2020 report issued by the World Health Organization, the global nursing workforce faces a deficit of approximately 5.9 million nurses, with inequitable distribution particularly evident in low- and lower-middle-income countries [2]. Subsequent international reports further emphasize that these shortages continue to threaten the achievement of universal health coverage and the Sustainable Development Goals [3, 4].

In exploring strategies to alleviate nursing shortages, professional identity has been widely recognized as a key factor influencing nurses’ career choice and retention [5]. Strengthening professional identity has been shown to reduce turnover intention and contribute to workforce stability [6]. As future members of the nursing workforce, nursing students’ professional identity is closely associated with both workforce supply and the quality of nursing care [7, 8]. Empirical evidence indicates that students with stronger professional identity are more likely to enter and remain in the nursing profession and demonstrate higher levels of professional behavior in clinical practice [9–11]. Therefore, enhancing professional identity among undergraduate nursing students is essential for ensuring workforce stability and promoting high-quality nursing care.

The university stage is a critical period for career exploration and professional value formation. During this stage, nursing education plays an essential role in shaping students’ understanding and development of professional identity. As a discipline integrating both natural sciences and human sciences, nursing education extends beyond the formal curriculum to diverse educational contexts, including institutional regulations, campus culture, learning climate, clinical practice arrangements, and everyday interactions. These contexts operate in implicit and often subtle ways, exerting a pervasive influence on students’ knowledge, emotions, beliefs, and behaviors [12, 13]. This implicit influence, which is not explicitly incorporated into formal teaching plans or curricular objectives but conveys values, norms, and professional meanings through everyday educational practices, is referred to as the hidden curriculum [14, 15]. Previous studies have demonstrated that the hidden curriculum plays a significant role in the development of nursing students’ professional identity. A positive and supportive hidden curriculum may enhance nursing students’ professional ethics and professional behaviors and may also strengthen their self-efficacy and professional identity [16, 17]. Conversely, when implicit messages conflict with students’ professional expectations, negative consequences such as reduced empathy, weakened professionalism, and diminished professional identity may occur [18, 19].

In addition to its potential direct influence on professional identity, the hidden curriculum may also indirectly promote professional identity development by shaping students’ learning engagement. Learning engagement refers to the time, energy, and emotional resources that students invest in the learning process [20]. Studies have shown that key components of the hidden curriculum, including the learning environment, institutional regulations, and student–teacher relationships, are important factors associated with students’ learning engagement [21, 22]. Evidence also suggests that the hidden curriculum may play a positive role in stimulating students’ interest in learning and enhancing their level of engagement [23]. Furthermore, higher levels of learning engagement have been found to be closely related to stronger professional identity. A study conducted by Song et al. among teacher education students reported that for every one-unit increase in learning engagement, professional identity increased by approximately 0.3 units [24]. Related review studies have similarly indicated that greater engagement is associated with positive professional development outcomes, including professional behaviors, professional commitment, and the quality of nursing care [25].

In summary, existing evidence suggests potential interrelationships among the hidden curriculum, learning engagement, and professional identity. However, prior research has predominantly focused on pairwise associations between these variables, and integrative analyses of their combined mechanisms remain limited. In particular, among undergraduate nursing students, the potential mediating role of learning engagement in the relationship between the hidden curriculum and professional identity has not been sufficiently explored. Therefore, this study aims to examine the specific pathways through which the hidden curriculum may influence professional identity among undergraduate nursing students, with the goal of providing theoretical insights and practical implications for optimizing nursing education strategies and enhancing students’ professional identity.

## Theoretical framework

This study is grounded in Bandura’s theory of triadic reciprocal determinism as its theoretical framework [26], which constitutes a core component of social cognitive theory and emphasizes the relationships among personal factors, behavioral processes, and environmental influences. Specifically, personal factors refer to individuals’ internal characteristics, such as cognition and emotions; behavior denotes individuals’ actions and performances in specific contexts; and environmental factors encompass external conditions, including social, cultural, and educational influences. These three components influence one another and jointly shape individual development.

Within the framework of the present study, professional identity represents the personal factor dimension and is conceptualized as an internal cognitive structure encompassing students’ understanding of the nursing profession, emotional evaluations, and the internalization of professional values. The hidden curriculum represents the environmental factor dimension, encompassing implicit norms and value orientations conveyed through pathways such as campus culture, student–teacher interactions, and the learning climate within nursing education contexts. Learning engagement represents the behavioral dimension, reflecting the extent to which students devote time, emotional resources, and energy to learning activities.

According to the theory of triadic reciprocal determinism, the hidden curriculum, as an important environmental factor, may influence students’ learning engagement. Moreover, engagement in learning activities may shape and deepen students’ internal cognition of and emotional attachment to the nursing profession, namely professional identity. Therefore, the present study focuses on one potential pathway within this theoretical framework, proposing that the hidden curriculum (environmental factor) influences the professional identity of undergraduate nursing students (personal factor) through learning engagement (behavior). This pathway provides a theoretical foundation for a deeper understanding of the mechanisms underlying the formation of professional identity.

**Fig. 1:**
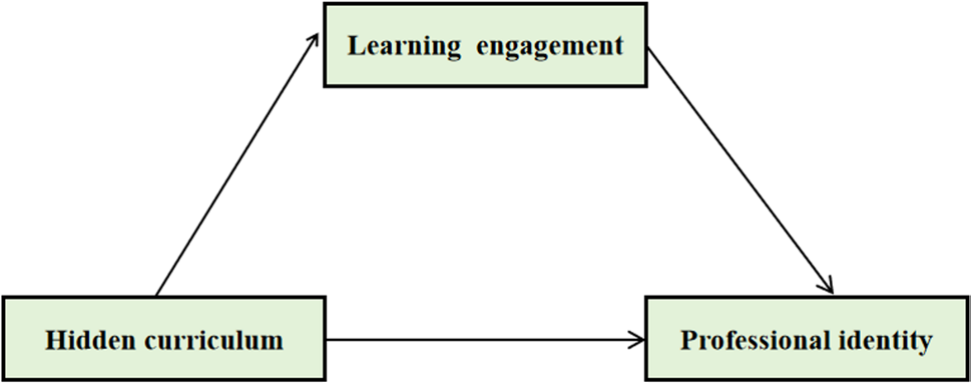
The hypothesized model

Based on the above theory and research, the hypothesis model of this study is shown in Fig. 1:

### Hypothesis 1

Hidden curriculum is positively associated with professional identity of undergraduate nursing students;

### Hypothesis 2

Hidden curriculum is positively associated with learning engagement of undergraduate nursing students;

### Hypothesis 3

Learning engagement is positively associated with professional identity of undergraduate nursing students.

### Hypothesis 4

Learning engagement plays a mediating role between hidden curriculum and professional identity of undergraduate nursing students.

### Methods

#### Study design

A cross-sectional study was conducted from November to December 2024.

### Participants

This study adheres to the STROBE guidelines for observational research and employed a convenience sampling method to survey full-time undergraduate nursing students from two public undergraduate medical universities in Hangzhou, Zhejiang Province, China. Both institutions are government-funded universities that offer standardized undergraduate nursing programs, and their educational structures, curriculum systems, training objectives, and teaching management models generally adhere to national standards for undergraduate nursing education, demonstrating a high degree of consistency in professional construction. The inclusion criteria were as follows: (1) Full-time undergraduates from freshman to the senior year of nursing; (2) Students who understand the purpose of this survey and agree to participate. Exclusion criteria: students who have taken a break from their studies or whose studies have been interrupted for other reasons. This study used the formula for calculating sample size in cross-sectional studies: N = (Zα²*σ²)/δ² [27], where the standard deviation was 11.90 (the standard deviation was estimated based on a prior study with a comparable population and identical measurement instrument) [28], the allowable error δ = 1, and the bilateral α = 0.05. Considering a potential dropout rate of 10 to 20%, the minimum sample size required was 599. A total of 667 questionnaires were distributed, and 623 valid questionnaires were collected, with a valid questionnaire collection rate of 93.4%.

### Data collection

After obtaining the approval of the school where the research participants were located, the researcher and trained assistants administered the paper-based questionnaires in person through on-site face-to-face surveys. Prior to data collection, the researchers provided participants with standardized instructions introducing the purpose and content of the study and emphasizing anonymous completion, voluntary participation, and confidentiality of the data. The participants completed the questionnaires independently. During the completion process, if participants had questions regarding the item wording, the researchers offered only standardized and neutral clarifications (e.g., explaining the literal meaning of items), without providing any prompts or guidance related to the content of responses. Upon completion, the questionnaires were collected on-site and checked for completeness. The exclusion criteria for questionnaires were as follows: (1) the presence of missing items or omissions; (2) evidence of clearly low-quality responses, such as straight-line or mechanically repetitive answering across a large number of items, or obvious logical inconsistencies with reverse-coded items.

### Instruments

#### Sociodemographic questionnaire

The sociodemographic questionnaire was self-designed by the researchers (Supplementary Material 1). The questionnaire included seven variables, including gender, grade, place of residence, whether the student was an only child, whether the student serves as a class cadre, and occupation-related questions (reasons for choosing nursing and relatives working in the nursing profession).

#### Hidden curriculum evaluation scale in nursing education (HCES-N)

In this study, the Chinese version of the HCES-N, originally developed by Akçakoca [29] and subsequently translated and culturally adapted into Chinese by Li et al. [30], was used to assess undergraduate nursing students’ perceptions of the hidden curriculum. The Cronbach’s α of the Chinese version reported in the validation study was 0.945. The HCES-N comprises three dimensions: school atmosphere, professional acquisitions, and student–teacher–school interaction, with a total of 44 items. All items are rated on a 5-point Likert scale ranging from 1 (“Never”) to 5 (“Always”). Items 34 to 44 within the dimension of student–teacher–school interaction are reverse-scored (“Always” = 1, “Often” = 2, “Sometimes” = 3, “Rarely” = 4, and “Never” = 5). The total score of the HCES-N ranges from 44 to 220, with higher scores indicating a more positive evaluation of the hidden curriculum in nursing education. In the present study, the total Cronbach’s α of the HCES-N was 0.961, and the dimension-specific Cronbach’s α coefficients ranged from 0.929 to 0.938. The Kaiser–Meyer–Olkin (KMO) value was 0.966, and Bartlett’s test of sphericity was significant (*P* < 0.001), indicating good reliability and construct validity of the scale in this sample.

#### Utrecht work engagement scale-student (UWES-S)

In this study, the Chinese version of the UWES-S was used to measure the level of learning engagement among nursing students. The Chinese UWES-S is an adapted instrument revised by Li et al. [31] on the basis of the learning engagement scale (UWES-S) originally developed by Schaufeli et al. [32], and has been modified to suit Chinese university students. The Cronbach’s α for the Chinese version of the UWES-S was 0.919. The scale consists of 17 items in 3 dimensions: motivation (6 items), vigor (6 items), and absorption (5 items). The score of each item ranges from 1 (“never”) to 7 (“always”). The scale scores are the sum of all items. The total score ranges from 17 to 119. A higher score indicates a higher level of learning engagement among nursing students. In this study, the total Cronbach’s α of the scale was 0.958, with dimension-specific Cronbach’s α values ranging from 0.879 to 0.906. The KMO value was 0.967, and Bartlett’s test of sphericity was significant (*P* < 0.001), indicating that the scale exhibited good reliability and validity in this study.

#### Professional identity scale for nursing students (PISNS)

The Professional Identity Scale for Nursing Students (PISNS) was used to assess professional identity among nursing students. It is a self-developed instrument designed by Chinese nursing researchers Hao et al. [33], and Cronbach’s α was 0.827. The scale includes five dimensions: professional self-image, benefits of retention and risk of turnover, social comparison and self-reflection, independence of career choice, and social modeling, with a total of 17 items. All items are rated on a 5-point Likert scale from 1 (Very Inconsistent) to 5 (Very Consistent), with item 12 being reverse-scored. The total score ranges from 17 to 85, with higher scores indicating a higher level of professional identity among nursing students. In this study, the Chinese version of the PISNS demonstrated high internal consistency, with a total Cronbach’s α of 0.919 and dimension-specific α coefficients ranging from 0.777 to 0.898. Validity testing yielded a KMO value of 0.952, and Bartlett’s test of sphericity was significant (*P* < 0.001), indicating that the scale possessed good reliability and validity within the context of this research.

### Data analysis

IBM SPSS 26.0 software and PROCESS macros (Model 4) were used for data analysis [34]. All continuous variables were tested for normality. Frequency and percentage were used to describe the sociodemographic characteristics. Independent t-tests and one-way ANOVA were conducted to examine differences in learning engagement and professional identity across sociodemographic variables. When the ANOVA results were significant, post hoc comparisons were performed using the LSD test. Sociodemographic variables significantly associated with professional identity and/or learning engagement were entered as covariates in the regression and mediation analyses. Pearson correlation analysis was used to explore the relationships among hidden curriculum, learning engagement, and professional identity. A mediation model (PROCESS Model 4) was conducted to test whether learning engagement mediates the relationship between hidden curriculum and professional identity. The bootstrap method with 5,000 resamples was applied, and a mediation effect was considered significant if the 95% confidence interval did not include zero. A p-value < 0.05 was considered statistically significant.

## Results

### Sociodemographic characteristics of participants

A total of 623 nursing undergraduates participated in this study. The majority of participants were female (77.2%); 19.4% were freshmen, 29.1% were sophomores, 34.8% were juniors, and 16.7% were seniors. 36.4% of the students were from urban areas. 30.5% of the students were only children in their families. 16.7% of the students chose nursing due to personal interest. 38.4% of the students had relatives working in the nursing profession. Table 1 displays more detailed participant characteristics.

**Table 1:** Socio-demographic characteristics of participants (*N* = 623)

Gender	Frequency (*n*)	Percentage (%)
Male	142	22.8
Female	481	77.2
Grade		
Freshman	121	19.4
Sophomore	181	29.1
Junior	217	34.8
Senior	104	16.7
Residence		
Urban	227	36.4
Rural	396	63.6
Only-child status		
Yes	190	30.5
No	433	69.5
Reasons for choosing nursing		
Personal interest	104	16.7
Advice from parents or others	297	47.7
Adjusted admission	81	13.0
Other	141	22.6
Serving as a class cadre		
Yes	205	32.9
No	418	67.1
Relatives work in the nursing profession		
Yes	239	38.4
No	384	61.6

Adjusted admission refers to students being assigned to the nursing major because their admission scores did not meet the requirements of their preferred major

### Social-demographic differences in learning engagement and professional identity

Differences in learning engagement and professional identity across sociodemographic characteristics are presented in Table 2. Significant grade differences were observed in learning engagement (*F* = 3.92, *P* < 0.01) and professional identity (*F* = 4.93, *P* < 0.01). LSD tests showed that senior students scored significantly higher than freshmen and sophomores in learning engagement. LSD tests showed that sophomores, juniors, and seniors all scored significantly higher than freshmen, while no significant differences were observed among the three upper grades.

**Table 2:** Social-demographic differences in learning engagement and professional identity (*N* = 623)

Variables		Learning engagement scores (M ± SD)	*t/F*	LSD test	Professional identity scores (M ± SD)	*t/F*	LSD test
Gender			1.04			1.31	
	Male	80.12 ± 15.49			62.85 ± 10.47		
	Female	78.60 ± 15.31			61.55 ± 10.43		
Grade			3.92^**﻿^	④ > ① ④ > ②		4.93^**﻿^	③ > ① ② > ① ④ > ①
	①Freshman	76.02 ± 14.16			58.74 ± 8.95		
	②Sophomore	77.90 ± 15.38			61.93 ± 9.85		
	③Junior	79.71 ± 15.81			63.06 ± 11.25		
	④Senior	82.57 ± 14.99			62.80 ± 10.75		
Residence			0.67			1.82	
	Urban	79.49 ± 15.31			62.85 ± 10.78		
	Rural	78.63 ± 15.38			61.27 ± 10.22		
Only-child status			-1.38			-0.67	
	Yes	77.66 ± 16.60			62.27 ± 10.47		
	No	79.51 ± 14.75			61.66 ± 10.43		
RCN			6.75^***﻿^	② > ④ ① > ② ① > ④		20.96^***﻿^	③ > ④ ② > ④ ① > ④ ① > ③ ① > ②
	①Personal interest	84.08 ± 13.90			68.11 ± 8.22		
	②APO	78.75 ± 15.26			61.77 ± 9.37		
	③Adjusted admission	79.41 ± 16.99			60.79 ± 11.39		
	④Other	75.30 ± 14.63			57.99 ± 11.43		
CLO			-4.32^***﻿^			-2.83^**﻿^	
	Yes	82.80 ± 16.00			63.53 ± 10.48		
	No	77.06 ± 14.67			61.02 ± 10.34		
RWNP			2.71^**﻿^			2.19^*﻿^	
	Yes	81.05 ± 15.73			63.01 ± 10.46		
	No	77.64 ± 14.98			61.13 ± 10.38		

Adjusted admission refers to students being assigned to the nursing major because their admission scores did not meet the requirements of their preferred major

*RCN* reasons for choosing nursing, *APO* advice from parents or others, *CLO* serving as a class cadre, *RWNP* relatives working in the nursing profession

* *P* < 0.05; ** *P* < 0.01; *** *P* < 0.001

Significant differences were also observed across reasons for choosing nursing (RCN) in learning engagement (*F* = 6.75, *P* < 0.001) and professional identity (*F* = 20.96, *P* < 0.001). LSD tests indicated that students who chose nursing based on personal interest scored significantly higher on both learning engagement and professional identity than those influenced by parents or others and those who selected “other reasons.” In addition, for professional identity, students in the “other reasons” group scored significantly lower than the other groups. Detailed results are shown in Table 2.

### Scores of undergraduate nursing students’ hidden curriculum, learning engagement, and professional identity

As shown in Table 3, the mean score of professional identity among nursing students was 61.85 (SD = 10.45). The mean scores of hidden curriculum and learning engagement were 176.98 (SD = 21.64) and 78.94 (SD = 15.35), respectively.

**Table 3:** Descriptive statistics and correlation analysis of three variables

Variable	Score range	Mean	SD	*r*		
				1	2	3
1. Hidden curriculum	44–220	176.98	21.64	—		
2. Learning engagement	17–119	78.94	15.35	0.54**	—	
3. Professional identity	17–85	61.85	10.45	0.58**	0.66**	—

** *P* < 0.01

The mean scores of the five dimensions of professional identity ranked from high to low were as follows: Social comparison and self-reflection (mean = 3.93, SD = 0.65), Social modeling (mean = 3.86, SD = 0.81), Professional self-image (mean = 3.60, SD = 0.75), Independence of career choice (mean = 3.48, SD = 0.72), and Benefit of retention and risk of turnover (mean = 3.45, SD = 0.79). Detailed results are presented in Table 4.

**Table 4:** Scores of the dimensions of hidden curriculum, learning engagement, and professional identity

		Number of items	Minimum	Maximum	Mean (Standard deviation)	Rank
Hidden curriculum	School atmosphere	21	2.24	5.00	3.89 (0.53)	3
	Professional acquisitions	12	2.00	5.00	4.06 (0.58)	2
	Student-teacher-school interaction	11	1.09	5.00	4.24 (0.63)	1
Learning engagement	Motivation	6	2.00	7.00	4.88 (0.91)	1
	Vigor	6	1.33	7.00	4.48 (1.00)	3
	Absorption	5	2.00	7.00	4.56 (0.96)	2
Professional identity	Professional self-image	6	1.00	5.00	3.60 (0.75)	3
	Benefit of retention and risk of turnover	4	1.00	5.00	3.45 (0.79)	5
	Social comparison and self-reflection	3	1.00	5.00	3.93 (0.65)	1
	Independence of career choice	2	1.00	5.00	3.48 (0.72)	4
	Social modeling	2	1.00	5.00	3.86 (0.81)	2

### Correlations among hidden curriculum, learning engagement, and professional identity

Table 3 presents the Pearson correlation coefficients among hidden curriculum, learning engagement, and professional identity. All three variables were significantly and positively correlated (*P* < 0.01), with correlation coefficients ranging from 0.54 to 0.66.

**Fig. 2:**
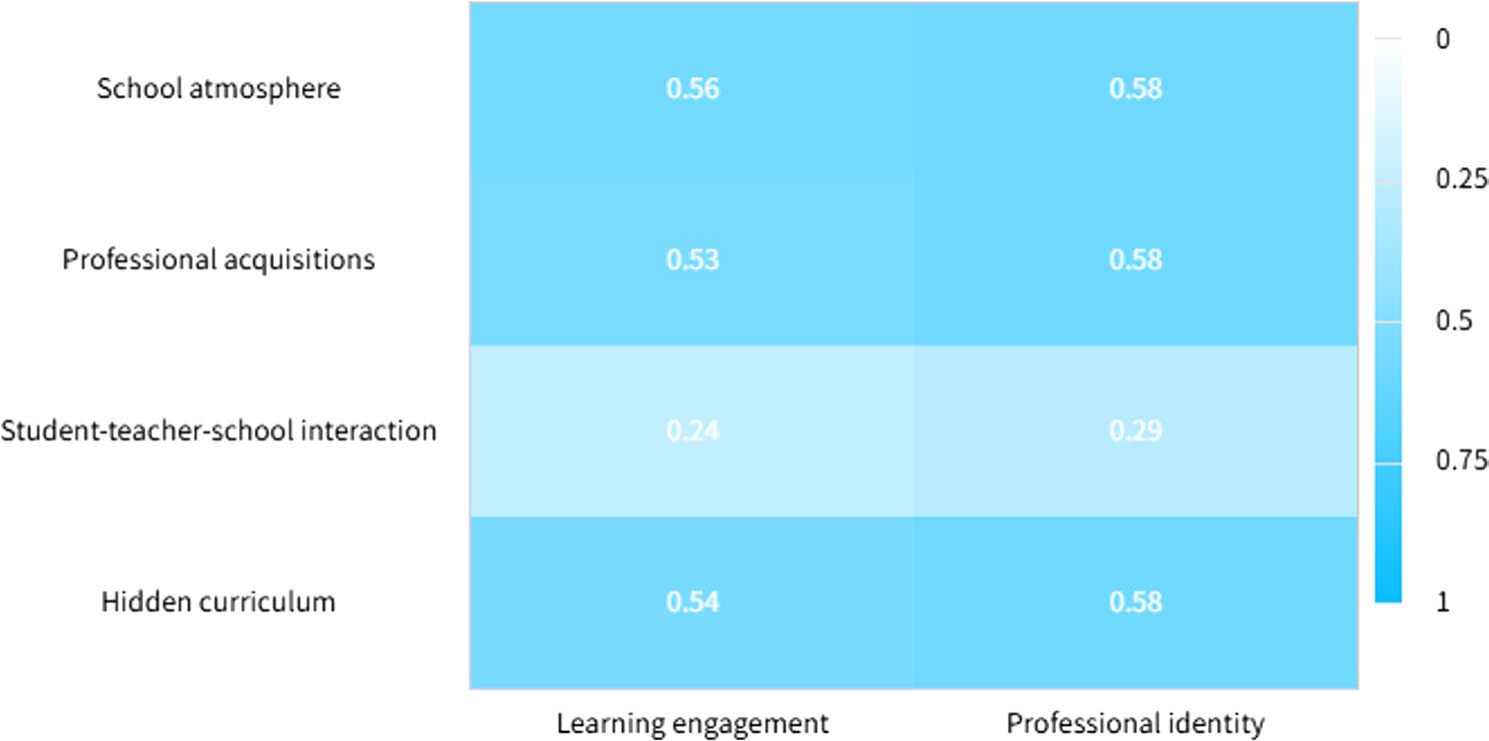
Correlations between the dimensions of the hidden curriculum and learning engagement and professional identity

To further examine the associations between the dimensions of the hidden curriculum and the two outcome variables, additional correlation analyses were conducted. As shown in Fig. 2, school atmosphere, professional acquisitions, and student–teacher–school interaction were all significantly and positively correlated with learning engagement (*r* = 0.56, 0.53, and 0.24, respectively; all *P* < 0.01) and professional identity (*r* = 0.58, 0.58, and 0.29, respectively; all *P* < 0.01).

### The results of regression and mediating analyses

Table 5 presents the regression results for the relationships among the study variables. After controlling for grade, reasons for choosing nursing, class leadership status, and whether students had relatives working in the nursing profession, the hidden curriculum was positively associated with professional identity among undergraduate nursing students (B = 0.263, *t* = 17.178, *P* < 0.001), supporting Hypothesis 1. Hidden curriculum was also positively associated with learning engagement (B = 0.376, *t* = 15.943, *P* < 0.001). After learning engagement was included in the model, the association between hidden curriculum and professional identity remained statistically significant (B = 0.147, *t* = 9.165, *P* < 0.001), and learning engagement was positively associated with professional identity (B = 0.309, *t* = 13.369, *P* < 0.001), supporting Hypotheses 2 and 3.

**Table 5:** Regression analyses predicting learning engagement and professional identity (*N* = 623)

	Model 1			Model 2			Model 3		
	B	*t*	*P*	B	*t*	*P*	B	*t*	*P*
Grade (ref. Freshman)									
Sophomore	1.067	1.111	0.267	−1.229	−0.832	0.406	1.447	1.710	0.088
Junior	1.708	1.835	0.067	0.349	0.244	0.807	1.600	1.952	0.051
Senior	1.875	1.712	0.087	3.658	2.173	0.030	0.744	0.768	0.443
RCN (ref. Personal interest)									
APO	−5.426	−5.832	< 0.001	−4.012	−2.805	0.005	−4.185	−5.077	< 0.001
Adjusted admission	−5.608	−4.617	< 0.001	−1.891	−1.013	0.311	−5.023	−4.693	< 0.001
Other	−7.559	−7.052	< 0.001	−4.333	−2.630	0.009	−6.219	−6.552	< 0.001
CLO (ref. Yes)									
No	−1.880	−2.669	0.008	−5.562	−5.137	< 0.001	−0.160	−0.252	0.801
RWNP (ref. Yes)									
No	−0.666	−0.985	0.325	−1.895	−1.824	0.069	−0.080	−0.134	0.894
Hidden curriculum	0.263	17.178	< 0.001	0.376	15.943	< 0.001	0.147	9.165	< 0.001
Learning engagement							0.309	13.369	< 0.001
R^2^	0.406			0.350			0.541		
f²	0.683			0.538			1.179		
F	46.637			36.751			72.015		
P	< 0.001			< 0.001			< 0.001		

*B* is unstandardized coefficient

Model 1: Hidden curriculum predicts professional identity; Model 2: Hidden curriculum predicts learning engagement; Model 3: Hidden curriculum and learning engagement jointly predict professional identity

f² represents the effect size calculated based on Cohen's formula f² = R²/(1−R²)

f² for Model 3 reflects the combined effect of all predictors, including the mediator; the incremental f² attributable specifically to learning engagement as mediator is Δf² = 0.294

Model 1 explained 40.6% of the variance in professional identity (R² = 0.406), corresponding to a large effect size (f² = 0.683). Model 2 explained 35.0% of the variance in learning engagement (R² = 0.350), corresponding to a large effect size (f² = 0.538). After including learning engagement, Model 3 explained 54.1% of the variance in professional identity (R² = 0.541), corresponding to a large effect size (f² = 1.179). The inclusion of learning engagement increased the explained variance by 13.5% (ΔR² = 0.135), yielding an incremental effect size of Δf² = 0.294.

**Fig. 3:**
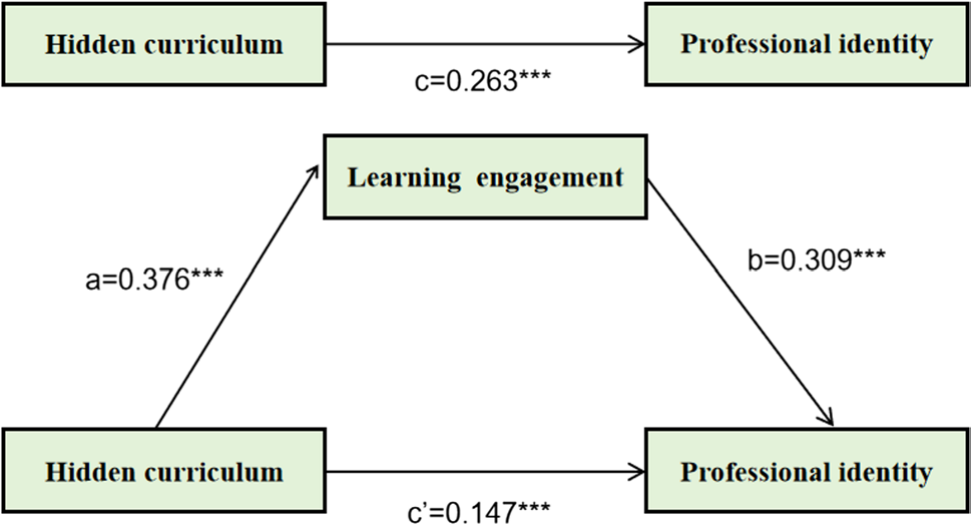
Mediation model of learning engagement in the relationship between hidden curriculum and professional identity. ***P < 0.001

The bootstrap 95% confidence intervals for the direct effect [0.116–0.179] and indirect effect [0.092–0.140] did not include zero, indicating a statistically significant mediation effect. The indirect pathway accounted for 44.13% of the total effect. Detailed results are shown in Table 6, and the mediation model is illustrated in Fig. 3. 

**Table 6:** Mediation effects of learning engagement on the association between hidden curriculum and professional identity (*N* = 623)

Effect type	Path	Effect ($\:\mathrm{B}$)	Boot SE	95% CI		PM
				LLCI	ULCI	
Total effect	Hidden curriculum→Professional identity (c)	0.263	0.015	0.233	0.293	—
Direct effect	Hidden curriculum→Professional identity (c’)	0.147	0.016	0.116	0.179	—
Indirect effect	Hidden curriculum→Learning engagement→Professional identity (a × b)	0.116	0.012	0.092	0.140	44.13%

*LLCI* lower limit of the confidence interval, *ULCI* upper limit of the confidence interval, *PM * proportion mediated, representing the proportion of the total effect explained by the indirect pathway

## Discussion

### Hidden curriculum, learning engagement and professional identity status quo

In this study, the total mean score of undergraduate nursing students’ evaluation of the hidden curriculum was (176.98 ± 21.64). Compared with the median total score of the HCES-N (132.00), this result suggests that the overall level of the hidden curriculum in nursing education was moderately high.

At the dimensional level, the student–teacher–school interaction dimension received the highest score (4.24 ± 0.63), followed by professional acquisitions (4.06 ± 0.58) and school atmosphere (3.89 ± 0.53). This ranking indicates that nursing students generally hold positive perceptions of interactions among students, teachers, and the school, as well as the educational environment. This may reflect the increasing emphasis on supportive teacher–student relationships and educational equity in contemporary nursing education. However, the relatively lower score for the school atmosphere dimension suggests that certain aspects of the broader educational environment may still require further improvement. One possible explanation is that some nursing educators may not have a sufficiently deep understanding of the concept of the hidden curriculum and its educational value; consequently, its educational functions may not be systematically and intentionally integrated into teaching practice. In addition, the lack of a unified and scientifically grounded evaluation framework for the hidden curriculum may limit the accurate assessment of its implementation and hinder the continuous improvement of nursing education.

In this study, undergraduate nursing students demonstrated a moderately high level of learning engagement (78.94 ± 15.35), which was higher than the median total score of the UWES-S (68.00). The total score was also higher than that reported among higher vocational nursing students [35]. This difference may be related to variations in educational level and learning context between the study populations.

At the dimensional level, the mean item scores ranked from highest to lowest as follows: motivation (4.88 ± 0.91), absorption (4.56 ± 0.96), and vigor (4.48 ± 1.00). This finding is consistent with the results reported by Li et al. [22]. Motivation is an important intrinsic driver of student learning and plays a key role in shaping learning behaviors. The relatively high score for motivation suggests that undergraduate nursing students generally recognized the value of the nursing profession and maintained a strong interest in learning. However, the relatively lower scores for vigor and absorption may indicate certain limitations in sustained persistence and concentration during learning. Previous research has shown that ineffective learning strategies may reduce learning efficiency and concentration [36]. Therefore, nursing educators should provide personalized guidance and support based on students’ individual needs and learning conditions. Through differentiated teaching approaches, educators can help nursing students identify learning methods that best suit them and thereby enhance their learning engagement.

In this study, the mean score of professional identity among undergraduate nursing students was (61.85 ± 10.45). This score indicated a moderately high level of professional identity compared with the median total score of the PISNS (51.00), and it was higher than the scores reported in pre-pandemic studies [11]. One possible explanation is that the COVID-19 pandemic increased public recognition of the nursing profession. During the pandemic, the professional dedication and social responsibility demonstrated by nurses received widespread recognition and extensive media coverage. Such social recognition may have enhanced nursing students’ perceptions of the value of the nursing profession and consequently strengthened their professional identity [37].

Among the dimensions of professional identity, the social comparison and self-reflection dimension showed the highest mean item score (3.93 ± 0.65). This finding is consistent with the results reported by He et al. [38] but differs from those of Guo et al., who reported this dimension as having the lowest score [39]. A possible explanation is that the expansion of social media and digital communication channels has increased nursing students’ exposure to societal evaluations and media representations of the nursing profession. Such exposure may enhance students’ awareness of social comparison and stimulate deeper reflection on their professional roles. Previous studies have also indicated that social recognition and support contribute positively to the development of professional identity among nursing students [40].

In contrast, the dimension of benefit of retention and risk of turnover had the lowest mean score (3.45 ± 0.79), which is consistent with the findings reported by Leng et al. [41]. This result may be related to the multiple external pressures currently faced by the nursing profession. In recent years, the tension in nurse–patient relationships and the increasing public attention to incidents of violence against healthcare workers have raised concerns among nursing students regarding the safety and social environment of nursing practice. In addition, previous research has shown that nurses often experience pressures related to salary levels, perceived income fairness, and imbalances between effort and reward. These factors may weaken nursing students’ expectations regarding future career rewards and development opportunities, thereby reducing their intention to remain in the nursing profession [42]. 

### Differences in learning engagement and professional identity across demographic characteristics

This study found that professional identity among undergraduate nursing students showed a gradual increase across academic years, followed by a slight decline in the senior year. This pattern may be related to changes in the learning context. In China, undergraduate nursing education typically requires students in their senior year to undertake clinical internships in teaching hospitals under the supervision of clinical instructors [43]. The transition from a campus-based learning environment to an authentic clinical setting exposes students to complex nursing tasks and higher levels of occupational stress. These challenges may prompt students to re-evaluate their professional identity. Previous studies have similarly shown that nursing students in the clinical internship stage are more likely to experience professional burnout and declines in professional identity, and some may even choose to leave the nursing profession after graduation [44]. In contrast, learning engagement increased progressively across academic years. LSD tests showed that seniors had significantly higher levels of learning engagement than freshmen and sophomores. This trend may reflect the gradual consolidation of professional knowledge and learning strategies as students advance through their studies.

Reasons for choosing the nursing major were significantly associated with both learning engagement and professional identity. LSD tests showed that students who chose nursing based on personal interest had significantly higher professional identity than those in all other groups. Intrinsic motivation may enhance students’ confidence in their professional competence and strengthen their emotional identification with the nursing profession. Students who chose nursing in response to their parents’ or others’ suggestions showed relatively higher professional identity than those who were admitted through major adjustment or for other reasons, while students admitted through major adjustment scored higher than those in the “other reasons” group. These findings suggest that different forms of passive entry into the nursing major may exert different degrees of influence on professional identity. Parental expectations often convey relatively positive perceptions of the stability and social value of the nursing profession, whereas students who entered nursing through major adjustment or for other ambiguous reasons may have had weaker initial career intentions.

A similar trend was observed for learning engagement. Students who chose nursing based on personal interest showed significantly higher learning engagement than those in the parents’ or others’ suggestion group and the “other reasons” group, while those in the parents’ suggestion group also scored higher than those in the “other reasons” group. This suggests that a clearer motivational basis for choosing nursing may be associated with higher levels of learning engagement.

Students who served as class leaders reported significantly higher levels of both learning engagement and professional identity. This may be because leadership roles provide more opportunities for interaction with teachers, greater exposure to positive professional information, and increased participation in school-organized activities, all of which may contribute to the development of professional identity. Similarly, having relatives working in the nursing profession was also significantly associated with both variables, which is consistent with previous research [45]. Long-term exposure to the values and experiences of healthcare professionals within the family environment may deepen students’ understanding of the nursing role and thereby strengthen their professional identity.

No significant differences were observed in learning engagement or professional identity among students of different genders, place of residence, or only-child status. One possible explanation is that undergraduate nursing programs in China generally follow relatively standardized curricula and training structures, meaning that students are exposed to broadly similar educational environments regardless of their demographic backgrounds. In addition, contemporary nursing education places increasing emphasis on equitable access to learning resources and opportunities, which may further reduce differences associated with demographic characteristics.

### Correlations among hidden curriculum, learning engagement, and professional identity

The results showed a significant positive correlation between the hidden curriculum and professional identity (*r* = 0.578, *P* < 0.01), which is consistent with Hypothesis 1 and aligns with the findings reported by Kaya et al. [46]. Professional identity is a core component of humanistic education and cannot be effectively transmitted through didactic instruction alone; instead, it requires positive guidance through the subtle and implicit influence of the hidden curriculum. According to social learning theory, the value orientations conveyed through teacher role modeling, professional behavioral norms in clinical practice, and the educational environment, all of which are reflected in the hidden curriculum, together constitute an important context for the professional socialization of nursing students. Through continuous observation, interaction, and situated experience, nursing students gradually internalize these professional values, thereby promoting the development of their professional identity [47].

Hidden curriculum was also significantly positively correlated with learning engagement among nursing students (*r* = 0.537, *P* < 0.01), supporting Hypothesis 2. Existing research indicates that a supportive and positive educational environment within schools is associated with enhanced student motivation and learning interest, both of which are recognized as key antecedents of learning engagement [48]. Nursing students who demonstrate higher levels of motivation are more inclined to participate actively in learning and exhibit stronger learning engagement [49].

Notably, differences were observed in the strength of associations between different dimensions of the hidden curriculum and both professional identity and learning engagement. Specifically, the dimensions of school atmosphere and professional acquisition showed relatively strong correlations with both professional identity (*r* = 0.58 and 0.58, respectively) and learning engagement (*r* = 0.56 and 0.53, respectively). In contrast, the student-teacher-school interaction dimension was more weakly associated with both outcome variables. From the perspective of social cognitive theory, this difference may reflect distinct mechanisms through which different dimensions influence professional development. The dimensions of school atmosphere and professional acquisition primarily involve direct and vicarious experiences related to professional value cognition, role understanding, and competence development. These experiences may enhance students’ self-efficacy and positive outcome expectations, thereby exerting a more direct influence on the formation of professional identity. By contrast, the student-teacher-school interaction dimension mainly reflects students’ perceptions of negative educational experiences, such as unfair treatment or suppressive interactions, and is measured using reverse-scored items. Therefore, this dimension may function more as a contextual safeguard for learning and identity development rather than as a direct driving factor. A fair and supportive educational environment helps sustain students’ learning engagement [50], whereas perceptions of unfair or suppressive interactions may hinder the development of learning behaviors and professional identity [51].

Learning engagement was significantly positively correlated with professional identity (*r* = 0.66, *P* < 0.01), supporting Hypothesis 3, and this finding is consistent with the results reported by Lin et al. [52]. When nursing students invest more time, energy, and emotional resources in learning, they are more likely to develop a deeper understanding of the value, meaning, and future prospects of the nursing profession, thereby laying a stronger cognitive and emotional foundation for the formation of professional identity. Conversely, students with a higher level of professional identity may also show greater motivation and persistence in learning. This interpretation is consistent with the findings of Liu et al., who reported that students with stronger professional identity tend to demonstrate higher levels of learning engagement [53].

### Mediating effect of learning engagement on the relationship between hidden curriculum and professional identity among undergraduate nursing students

The results showed that the hidden curriculum was an important predictor of professional identity among nursing students, and that learning engagement partially mediated the relationship between the hidden curriculum and professional identity, thereby supporting Hypothesis 4. This indicates that the hidden curriculum could influence nursing students’ professional identity not only directly, but also indirectly through the mediating role of learning engagement.

According to Bandura’s Triadic Reciprocal Determinism, individual development is shaped by the dynamic interplay among environmental, behavioral, and personal factors. The present findings suggest that, in the context of nursing education, the hidden curriculum, as an important environmental factor, may not only directly influence nursing students’ cognition and emotions toward the nursing profession, but may also further affect the development of professional identity by promoting their learning engagement. This mechanism may manifest differently across different stages of nursing education.

This mechanism may manifest differently across stages of nursing education. In the early stage of undergraduate study (the first and second years), students are gradually exposed to elements of the hidden curriculum through campus culture, teacher role modeling, and the implicit transmission of professional values. At this stage, professional identity is still developing and remains relatively malleable. By gradually increasing their engagement in learning, students may transform the implicit professional messages embedded in the educational environment into an initial understanding of the nursing profession, thereby facilitating the development of professional identity.

During the middle stage (the third year), students’ professional knowledge becomes increasingly systematic, and their level of learning engagement also rises accordingly. At this stage, learning engagement reflects not only students’ learning behaviors but may also promote a deeper understanding of professional roles, professional norms, and the value of the nursing profession, thereby further strengthening professional identity.

When students enter the clinical internship stage (the fourth year), the primary source of the hidden curriculum shifts from the campus-based learning environment to authentic clinical settings, including ward culture, institutional norms, and the professional behaviors demonstrated by clinical instructors. The clinical practice environment is usually more complex, requiring students to invest greater effort in adapting to new learning tasks, which may lead them to exhibit higher levels of learning engagement. However, compared with the campus environment, the clinical setting conveys more diverse information, including not only positive professional role models but also the real-world pressures of nursing practice. In this study, fourth-year students showed the highest level of learning engagement, yet their professional identity scores did not further increase compared with those of third-year students. This finding suggests that, during the clinical internship stage, even when students maintain a high level of learning engagement, the diverse and complex professional messages conveyed in the clinical environment may still directly influence their professional identity. This interpretation is also consistent with the present finding that learning engagement only partially mediated the relationship between hidden curriculum and professional identity.

### Implications for nursing education

The findings of this study provide important implications for nursing education. For lower-year students (the first and second years), professional identity is still in the formative stage, and students’ understanding of the nursing profession is usually limited. Therefore, educational practice may focus on helping students gradually recognize the implicit professional values embedded in the learning environment. For example, universities may provide early opportunities for professional exposure, such as organizing monthly group discussions, professional narrative-sharing sessions, or experience-sharing activities with clinical nurses. Through guided reflective activities, students can be encouraged to think about the roles and values of the nursing profession, thereby gradually developing an initial sense of professional identity.

For middle-year students (the third year), the educational focus may shift toward strengthening learning engagement through structured reflective learning. For example, after simulation training or clinical skills laboratory sessions, educators may guide students to reflect on the professional behaviors, communication strategies, and professional norms demonstrated in clinical cases. Writing reflective notes or participating in group discussions may further promote students’ understanding and internalization of professional values, enabling them to gradually transform learning experiences in the educational environment into sustained learning behaviors.

For senior nursing students in the clinical internship stage (the fourth year), who are directly exposed to complex clinical environments, universities and clinical training sites may strengthen collaborative management and provide support through a relatively stable clinical mentorship system. For example, during internship rotations, students may be assigned designated clinical instructors to provide practical guidance and regular feedback. In addition, organizing monthly peer reflection groups across different internship departments and proactively offering psychological support, especially for students who entered the program through passive pathways, may help maintain the stability of professional identity throughout the internship period.

### Limitations and future directions

This study has several limitations. First, the survey was conducted using convenience sampling in two medical universities in Hangzhou, which may limit the generalizability of the findings. Second, this study adopted a cross-sectional design and therefore cannot establish causal relationships among the variables, nor can it rule out potential reverse causal pathways. According to Triadic Reciprocal Determinism, dynamic interactions may exist among environmental, behavioral, and personal factors. For example, students with a higher level of professional identity may be more likely to attend to and perceive positive hidden curriculum elements in the educational environment and to demonstrate higher levels of learning engagement. In addition, individual factors such as self-efficacy and career expectations may also influence the relationships among the variables, which warrants further examination in future research.

Future studies may adopt longitudinal or experimental designs to further clarify the causal directions among these variables and their changes over time, and to explore moderating or interaction models incorporating both individual and environmental factors. Such efforts would further deepen the application of Triadic Reciprocal Determinism in the context of nursing education.

## Conclusion

Nursing workforce shortages have become a major challenge facing health systems worldwide. In this context, promoting nursing students’ professional identity through high-quality nursing education and reducing the risk of talent attrition are particularly critical. The present study found significant positive associations among the hidden curriculum, learning engagement, and professional identity, with learning engagement playing an important mediating role in the relationship between the hidden curriculum and professional identity. These findings suggest that nursing education practice may benefit from greater attention to the systematic optimization of the hidden curriculum, the cultivation of a positive educational environment, and the consideration of differentiated teaching strategies that promote learning engagement in accordance with the characteristics of students at different educational stages, thereby supporting the development of professional identity among nursing students.

## Supplementary Information

Supplementary Material 1.

## Data Availability

The data that support the findings of this study are available from the corresponding author upon reasonable request.
