# Supplementary material for: Beyond the formal curriculum: unveiling the pathway from hidden curriculum to professional identity through learning engagement in nursing education
Source: BMC Med Educ. 2026 May 2;26:995. doi: 10.1186/s12909-026-09332-2 (PMC13277233; doi:10.1186/s12909-026-09332-2)
Supplement: Supplementary file 1 — Supplementary Material 1. [file 12909_2026_9332_MOESM1_ESM.docx]

**Questionnaire**

This questionnaire was developed by the research team based on a review of relevant literature. It was used to collect demographic and study-related information for participants in the study.

1. Gender:

□ Male  □ Female

1. What is your current year of study (in university)?

□ Freshman  □ Sophomore  □ Junior  □ Senior

1. I originate from:

□ A rural area  □ An urban area

1. Only-child status:

□ Yes  □ No

1. Reasons for choosing nursing:

□ Personal interest  □ Advice from parents or others

□ Adjusted   □ Other reasons

1. Do you hold a position as a class officer? (e.g., class monitor, committee member, or other student leadership role):

□ Yes  □ No

1. Do you have any relatives working in the nursing profession?

□ Yes  □ No
